# Impact of oxycodone for the treatment of acute postoperative pain in cesarean section: A review

**DOI:** 10.1097/MD.0000000000041645

**Published:** 2025-02-21

**Authors:** Qingqing Pei, Hongmei Xuan, Zhiyou Peng

**Affiliations:** a Department of Anesthesiology, Beilun District People’s Hospital of Ningbo, Ningbo, China; b Department of Pain Medicine, Zhuji People’s Hospital of Zhejiang Province, Zhuji, China; c Department of Painology, The First Affiliated Hospital, Zhejiang University School of Medicine, Hangzhou, China.

**Keywords:** esarean section, oxycodone, patient-controlled intravenous analgesia, postoperative pain

## Abstract

The review aimed to summarize the recent pharmacological and published clinical trials that used oxycodone for pain management after cesarean section (CS). This narrative review is based on published studies in PubMed, EMbase, Web of science, and EBSCO on oxycodone for pain control after CS. Random studies that used oxycodone only or used oxycodone as a major part of a multimodal analgesia regimen were included. Non-English trials, abstract of conference, letters to the editor, animal studies, or studies with insufficient data were excluded. The initial search terms included a combination of free text words and Medical Subject Headings terms. There are 14 clinical trials included and the total number of participants was 1651. These included documents disputed oral oxycodone and patient-controlled intravenous analgesia (PCIA) morphine, compared oral oxycodone and intravenous morphine, investigated sustained-release oral oxycodone and intrathecal morphine, investigated slow release tapentadol and controlled-release oxycodone, investigated ketoprofen, combination of acetaminophen + oxycodone, acetaminophen, and placebo, evaluated oral oxycodone and epidural ropivacaine + sufentanil, evaluated oral oxycodone and PCIA piritramide, evaluated the combination oxycodone + acetaminophen and separately administered oxycodone/acetaminophen, compared the immediate-release oxycodone and controlled-release oxycodone, compared the oral and intravenous oxycodone, disputed PCIA oxycodone or morphine, compared epidural oxycodone and morphine, evaluated PCIA oxycodone, sufentanil or their combination. Oxycodone showed superior or similar postoperative analgesic efficacy compared with other opioids in various administration and reduced the need for rescue medication and side effects. Oxycodone can be successfully used for postoperative analgesia after CS with comparable side effects.

## 1. Introduction

Cesarean section (CS) is one of the most common surgical procedure performed in the world.^[1]^ The prevalence of CS has been increasing from year to year.^[2]^ In China, the CS rate reaches over 40% according to World Health Organization global survey.^[3]^ Adequate postoperative pain management is crucial, and it affects both the mother and child. Poor postoperative analgesia may adversely affect ambulation and breastfeeding, and increase complications such as venous thrombosis, pneumonia, and may even lead to persistent postsurgical pain.^[4–6]^ Ideally, postoperative pain management should provide adequate analgesia with minimal adverse effects. The use of opioids via different routes is the commonly used medications for pain relief after CS.^[7,8]^ They provide good pain control without decreasing touch reception, proprioception, and muscle power. The opioids include morphine, fentanyl, sulfentanil, hydromorphone, oxycodone, and so on.

Oxycodone is a semisynthetic opioid analgesic derived from the opium alkaloid thebaine, which has been recommended for the treatment of moderate to severe pain postoperatively by the World Health Organization. Oxycodone is a κ-opioid receptor agonist with a relatively low affinity for µ-opioid receptors.^[9,10]^ Clinical observations have shown that oxycodone was more effective than morphine in blocking visceral pain.^[11,12]^ It has been successfully used for postoperative analgesia for decades in various parts of the world such as Northern Europe and Canada.^[13]^ Different administration routes have been proposed for post-cesarean pain control, for example, intravenous, epidural, oral.^[14–16]^ For oral administration, oxycodone is available as immediate-release (IR) and controlled-release (CR) formulations.

The review aimed to summarize the recent pharmacological and published clinical trials that used oxycodone for pain management after CS.

## 2. Methods

Databases including PubMed, EMbase, Web of science, and EBSCO were searched to identify eligible articles published in the English language. The recommended references list of the retrieved studies were also reviewed to include other suitable studies. The initial search terms included a combination of free text words and Medical Subject Headings terms. The free text words included “(cesarean OR cesarean) AND oxycodone.” These search equation of PubMed was applicable to another database.

### 2.1. Inclusion criteria

Study type: random studies that used oxycodone only or used oxycodone as a major part of a multimodal analgesia regimen, administered postoperatively were included. The multimodal analgesia regimen included a combination of oxycodone and another form of analgesic, placebo, or other delivery routes of oxycodone such as intravenous administration and epidural injection. Patients: women who experienced CS and fulfilled the following standards, such as Anesthesiologists (ASA) physical status II, no hypertension, no cardiopulmonary disease, no severe pathological obstetrics, no history of alcohol, or substance abuse. Outcomes: analgesic efficacy (pain scores), the dosage of rescue medication, adverse effects, and postoperative recovery have reported in included studies.

### 2.2. Exclusion criteria

This review consisted of only published studies and excluded non-English trials, abstract of conference, letters to the editor, or animal studies, or studies with insufficient data.

## 3. Results

### 3.1. Search results and characteristics of clinical trials

**Figure 1.**
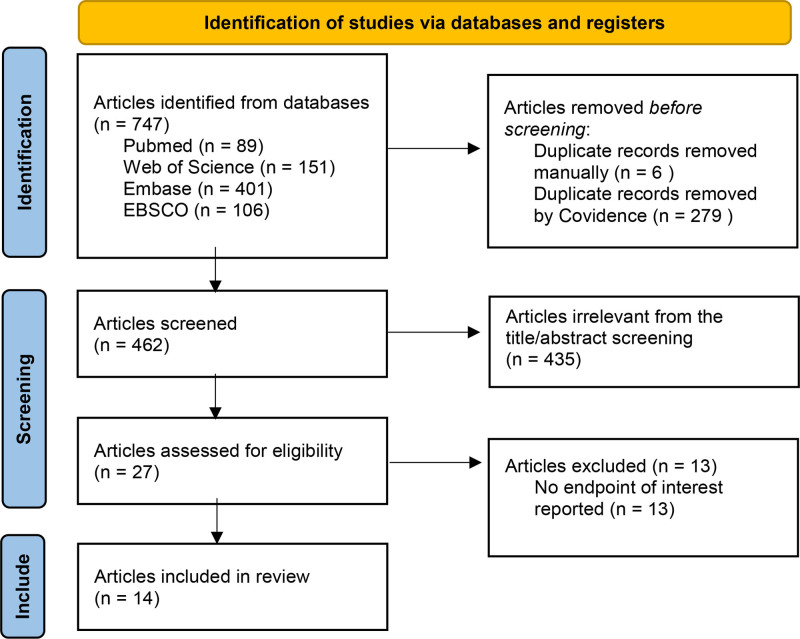
Flow diagram of the inclusion and exclusion process.

In accordance with the search strategy, 747 studies were included in the initial search on October 30, 2024. Of these, 285 articles were excluded due to duplicate literature. After reviewing the title and abstract, 435 articles were excluded because of irrelevant literature. A further 13 studies were then excluded because of a lack of intended intervention and conference abstracts. Finally, there were 14 clinical trials that fulfilled the inclusion criteria and were included in the review.^[14–27]^ The total number of participants for included studies was 1651. The study selection processes are shown in Figure 1. The characteristics of the 14 eligible studies in this review is shown in Table 1. The extracted data included first author, the published year, sample size, intervention, and outcomes measured. The doses and methods for the oxycodone and comparator were different in each study. Due to heterogeneity of different interventions, the included studies could not be pooled for meta-analysis. Therefore, narrative review is presented in this study. For all the included studies, statistical significance was defined as *P* < .05.

**Table 1:** Characteristics of clinical trials.

Author (year)	Study type	Duration of surgery (mean) min	Duration of study postop (h)	Drugs administered (n = number of participants)	Dosage and frequency	Rescue medication	Outcome
Kathryn et al (2006)	Prospective randomized study	Group oral oxycodone: 77.2 ± 29.5 group IV PCA morphine 62.2 ± 22.2	24 h	Group oral oxycodone: 5/325 mg oxycodone/acetaminophen (46) group IV PCA morphine: morphine (47)	Oral analgesia group: 2 tablets of 5/325 mg oxycodone/acetaminophen every 3 hours for first 12 hours, thereafter, 1 to 2 tablets every 4 hours as needed. group IV PCA morphine: 1 mg/h + 1 mg on demand for first 12 hours, thereafter, PCA discontinued and 1 to 2 oxycodone/acetaminophen tablets every 4 hours as needed	Meperidine (50 mg)	Postoperative pain: visual analog pain scale score Postoperative side effects/recovery: Nausea Drowsiness Pruritus Emesis Ambulation Oral intake
Max et al (2012)	Randomized controlled trial	Group oral oxycodone: 39.4 ± 13.3 group IV PCA piritramide: 38.3 ± 9.8	72 h	Group oral oxycodone: oral oxycodone 20 mg (113) group IV PCA piritramide: piritramide (126)	Group oral oxycodone: oral oxycodone 20 mg at 2 and 12 hours after CS group IV PCA piritramide: piritramide 2 mg/mL, 1 mg loading dose, lock out interval of 5 minutes, the maximum dose limited to 30 mg, discontinued at 24 hours	Ibuprofen acetaminophen	Postoperative pain: Visual analog pain scale score Hospital costs
Boel et al (2015)	Randomized open parallel group study	–	48 h	Group oral oxycodone: oral oxycodone (38) group IV morphine: IV morphine (39)	Group oral oxycodone: oral oxycodone 20 mg at 0 hours, 10 mg every 12 hours for minimum 48 hours. group IV morphine: 10 mg morphine for 24 hours; thereafter, 2 × 30 mg codeine every 6 hours for minimum 48 hours. Both treatment groups received supplemental ibuprofen/acetaminophen 48 hours after surgery.	Ibuprofen Acetaminophen	Postoperative pain: Numerical Rating Scale rescue medication Postoperative side effects/recovery: Stand next to the bed (h) Walking around with help (h) Fully mobilized (h) First bowel movement Discharge from hospital
McDonnell et al (2010)	Randomized, double-blind	–	24 h	Group oral oxycodone: oral oxycodone (55) group intrathecal morphine: intrathecal morphine 100 μg (56)	Group oral oxycodone: 20 mg SR at 0 hours; thereafter, 10 mg IR every 6 hours for 24 hours 100 µg morphine at spinal injection	IR oxycodone Tramadol	Postoperative pain: Numerical Rating Scale rescue medication Additional analgesia requested Postoperative side effects/recovery: Nausea Pruritus Satisfaction with analgesia Urinary retention Respiratory depression
Katja et al (2019)	Prospective randomized study	Group oral oxycodone: 33 group IV PCA oxycodone: 35	24 h	Group oral oxycodone: oral oxycodone (137) group IV PCA oxycodone: IV PCA oxycodone (133)	Group oral oxycodone: oxycodone/naloxone 10/5 mg, ibuprofen 600 mg, and paracetamol 1 g orally 1 hour after surgery. Oxycodone/naloxone 10/5 mg every 12 hours, and ibuprofen/paracetamol every 8 hours were given. group IV PCA oxycodone: oxycodone bolus doses of 2 mg and a lockout time of 10 minutes.	Oxycodone	Postoperative pain: Numerical Rating Scale rescue medication Additional analgesia requested Postoperative side effects/recovery: Nausea vomit Abdominal distension First meal Mobilization Defecation
Hj Wang et al (2019)	Prospective randomized study	–	48 h	Group IV PCA oxycodone: oxycodone (30) group IV PCA morphine: morphine (30)	0.8 mg/kg oxycodone or 0.8 mg/kg morphine using IV PCA device: diluted in 100 mL, 2 mL/h continuous dose, and a booster dose of 2 mL/15 minutes.	/	Postoperative Pain: Numerical Rating Scale
R. Ffrench et al (2019)	Randomized controlled trial	–	48 h	Group oral oxycodone: oral oxycodone 20 mg (35) group oral tapentadol: tapentadol 50 mg (33)	Group oral oxycodone: oral oxycodone 20 mg every 12 hours group oral tapentadol: tapentadol 50 mg every 12 hours	Oxycodone or tapentadol	Postoperative pain: the sum of pain intensity difference (SPID) total pain relief (TOTPAR), sum of total pain relief and pain intensity difference (SPRID) rescue medication Postoperative side effects/recovery: Itch Nausea Vomiting Shivering Drowsiness Dizziness Other side effects At least one side effect Absence of bowel motion within 48 hours post op
Ban et al. (2016)	Randomized controlled trial	Group epidural oxycodone: 45 group epidural morphine: 47	24 h	Group epidural oxycodone (50) group epidural morphine (50)	Group epidural oxycodone: 3 mg group epidural morphine: 3 mg	Tramadol	Postoperative pain: Numerical Rating Scale Postoperative side effects/recovery: pruritus Nausea Vomiting
Jing-jing et al. (2017)	Prospective, randomized, double-blind study	Group S: 53.8 ± 7.4 group OS1: 51.3 ± 4.3 group OS2: 52.9 ± 6.9 group O: 49.7 ± 7.6	24 h	Group S (sufentanil 100 μg) (30), group OS1 (sufentanil 70 μg, oxycodone 30 mg) (30), group OS2 (sufentanil 50 μg, oxycodone 50 mg) (28), and group O (oxycodone 100 mg) (29).	Drugs were diluted to 100 mL and managed with a continuous infusion of 1 mL/h, a bolus dose of 2 mL, and a lockout interval of 15 minutes. The maximum dose of patient-controlled intravenous analgesia per hour was 10 mL.	–	Postoperative pain: Numerical Rating Scale amount of opioid consumption Postoperative side effects/recovery: Hypotension Hypoxemia Respiratory depression Nausea Vomiting Pruritus Dizziness
Abraham et al. (1993)	Prospective, randomized, double-blind study	–	8 h	Group 100 mg ketoprofen (48) group 50 mg ketoprofen (48) group 650 mg acetaminophen combined 10 mg oxycodone hydrochloride (48) group 650 mg acetaminophen (48) group placebo (48)	Oral 100 mg ketoprofen, 50 mg ketoprofen, 650 mg acetaminophen combined 10 mg oxycodone hydrochloride, 650 mg acetaminophen, group placebo	Remedication with the same drug	Postoperative pain: The pain intensity difference (PID) score The sum of the pain intensity differences (SPID) TOTPAR: the sum of the hourly pain relief values
Zhong et al. (2014)	Randomized controlled trial	–	48 h	Group epidural analgesia (30) group oral oxycodone (30)	Patient-controlled epidural analgesia with 0.1% ropivacaine + 0.1 μg/mL sufentanil (for postoperative 48 hours) + injected pethidine on demand (E group); or CR oxycodone (2 × 15 mg for the first postoperative 24 hours; 2 × 10 mg for the second postoperative 24 hours) + paracetamol and tramadol hydrochloride tablets (8 × 1 tablet for the postoperative 48 hours) orally + injected pethidine on demand (O group).	Pethidine	Postoperative pain: visual analog scale pethidine consumption Postoperative side effects/recovery: Time to free mobilization (walking without assistance), hospital stay, nausea and vomiting, pruritus, dizzy, bowel function, maternal satisfaction
Courtney et al. (2022)	Retrospective cohort study	–	–	“Combined Medication” group (63) “Separate Medication” group (87)	The “Combined Medication” group: oxycodone 5 mg-acetaminophen 325 mg one or-two tabs Q4h prn. the “Separate Medication” group: n acetaminophen 650 mg Q4 h prn and oxycodone 5 mg or 10 mg Q4h prn. All parturient were prescribed ketorolac Q6h scheduled for 24 hours, and then ibuprofen 600 mg Q6h prn was followed.	–	Opioid medication use per 12-h period in intravenous morphine mg equivalents.
Schoenwald et al. (2018)	Randomized controlled trial	–	–	The IR oxycodone group (61) the CR oxycodone group (61)	IR oxycodone was given at 8:00 hours (10 mg), 14:00 hours (5 mg), and 20:00 hours (5 mg) on the day after surgery with nurse practitioner intervention. 10 mg of CR oxycodone was given at 08:00 hours and continuing per 12 h for 48 h. Oral paracetamol (one gram) Q6h was prescribed at 18:00 hours on the day of surgery, and oral ibuprofen (400 mg) QD for 72 h was also administrated at 08:00 hours on the first postoperative day.	Oxycodone or tramadol	Pain scores Total opioid doses
Aino Pesonen et al. (2024)	Randomized controlled trial	–	72 h	Controlled-release oxycodone/naloxone 10/5 mg tablets group (21) Controlled-release oxycodone 10 mg tablets group (22)	CR oxycodone/naloxone 10/5 mg tablets or CR oxycodone 10 mg tablets were administered twice a day at 8 am and 8 pm	Oxycodone	Pain scores Total opioid doses

CR = controlled-release, IR = immediate-release, PCA = patient-controlled analgesia, SR = sustained-release.

Oxycodone has wonderful oral bioavailability and long duration of action, thus making it a good choice for analgesia after CS. Some included studies compared the analgesic effectiveness of oral oxycodone with other analgesic drugs or other delivery routes/forms of oxycodone. These documents disputed the intravenous patient-controlled analgesia (IV PCA) morphine and oral oxycodone,^[22]^ compared oral oxycodone and intravenous morphine,^[23]^ investigated sustained-release oral oxycodone and intrathecal morphin,^[25]^ investigated slow release tapentadol and CR oxycodone,^[15]^ investigated ketoprofen, combination of acetaminophen + oxycodone, acetaminophen, and placebo,^[19]^ evaluated oral oxycodone and epidural ropivacaine + sufentanil,^[26]^ evaluated oral oxycodone and IV PCA piritramide,^[24]^ evaluated the combination oxycodone + acetaminophen and separately administered oxycodone/acetaminophen,^[21]^ compared the IR oxycodone and CR oxycodone,^[20]^ compared the oral and intravenous oxycodone,^[14]^ disputed IV PCA oxycodone or morphine,^[18]^ compared epidural oxycodone and morphine,^[17]^ evaluated IV PCA oxycodone, sufentanil or the combination of oxycodone + sufentanil,^[16]^ compared CR oxycodone/naloxone 10/5 mg tablets and CR oxycodone tablets.^[27]^

### 3.2. Postoperative pain

Studies had showed that the oral oxycodone could provide good analgesia postoperatively. In one study by Kathryn et al,^[22]^ oral oxycodone appeared to be more efficient in pain control after CS. In this study, researchers disputed the IV PCA morphine and oral oxycodone for analgesia after surgery. The PCA group received morphine according to the following protocol: 1 mg/h continuous dose, 1 mg self-controlled dose, 6 minutes lock interval, discontinued at 12 hours. Then oxycodone–acetaminophen (5/325 mg), with 1 to 2 tablets permitted Q4h for 12 hours as needed for pain relief. The oral oxycodone group immediately took 2 tablets of oxycodone–acetaminophen (5/325 mg), followed by 2 tablets of oxycodone–acetaminophen were administered Q3h for 12 hours, then 1 to 2 tablets were permitted Q4h prn. Controlled maximum dose was 12 tablets in 24 hours. As part of the multimodal analgesic regimen, patients in both groups were injected ketorolac 30 mg immediately postoperatively, followed by 15 mg Q6h for 24 hours. The oral oxycodone group reported less pain at 6 and 24 hours than the PCA group (*P* < .05). In another study by Aino Pesonen,^[27]^ CR oxycodone/naloxone 10/5 mg tablets or CR oxycodone 10 mg tablets were given to delivery women twice a day for the first 3 days after elective cesarean surgery. Both groups provided good pain relief. The pain scores were similar in the 2 groups, except that the most pain at wound compression on the surgery day and the pain at rest on postoperative day 3 were lower in CR oxycodone group.

Similarly, Niklasson et al found in their study that oral oxycodone was more efficient in providing a better analgesic effect than intravenous morphine.^[23]^ Firstly, all women took oral ibuprofen 400 mg before leaving the operating room, and continuously received 200 mg ibuprofen Q6h. Then all women randomly received oral oxycodone or intravenous morphine. Immediately after surgery, the oral oxycodone group received 20 mg long-acting oxycodone. Thereafter, 10 mg oxycodone was given Q12h for at least 48 hours. If analgesia was not enough, oral dose of 5 mg IR oxycodone was provided for rescue medication, intravenous 1 to 5 mg IR oxycodone was injected for breakthrough pain. 1 g oral paracetamol Q6h until discharged was offered in this group. The intravenous morphine group, 10 mg morphine was nurse-administered by slowly injected until the numerical rating scale was gained for the first 24 hours. Then, 2 tablets of paracetamol 500 mg plus codeine 30 mg Q6h was offered. Mean pain scores at rest at 0 to 6 hours, 25 to 48 hours, provoked pain (uterus palpation) at 0 to 6 hours, maximum pain intensity (when asking for rescue medication) at 0 to 24 hours, and number of rescue medications at 0 to 24 hours were less in the oxycodone group.

In another study by McDonnell et al,^[25]^ parturient were randomized to accept either sustained-release oral oxycodone 20 mg immediately after surgery followed by IR oxycodone 10 mg Q6h for 24 hours or preservative-free intrathecal morphine 100 μg at spinal injection. All parturient were provided intravenous parecoxib 40 mg postoperatively and oral diclofenac 50 mg Q8h starting at 12 hours after surgery. Immediate-release oral oxycodone 10 to 15 mg was administered for supplemental postoperative analgesia. If the numerical rating scale > 6/10, oral tramadol 100 mg was offered. Numerical pain scores were higher in oxycodone group. Only 35 patients requested for additional analgesia in morphine group, but 46 patients requested for additional pain-killing drugs in oxycodone group (*P* < .05). Parturient in oxycodone group more frequently reported high worst pain scores (pain scores 4–10, 36 vs 48, *P* < .05).

In the study by R. Ffrench et al,^[15]^ randomized patients were treated with either slow release tapentadol 50 mg or equivalent CR oxycodone 10 mg per 12 hours for 48 hours. All women received regular paracetamol and diclofenac postoperatively. If analgesia was inadequate, additional oxycodone 5 to 10 mg or tapentadol 50 mg was ordered. SPID48 means the sum of pain intensity difference (SPID) over the first 48 hours of treatment. Higher values of SPID presenting greater pain control. There was no significant difference in SPID48 between groups. The oxycodone group indicated higher SPID36 scores than tapentadol group with increased time to rescue drug. Patients in the oxycodone group reported a significantly increased time to demand for rescue analgesia compared to the tapentadol group (*P* < .05).

In Abraham et al study,^[19]^ women were randomly assigned into one of the 5 groups to receive: 100 mg ketoprofen, 50 mg ketoprofen, 650 mg acetaminophen combined 10 mg oxycodone hydrochloride, 650 mg acetaminophen, or placebo over an 8-hour period after surgery. The acetaminophen plus oxycodone group and 100 mg ketoprofen group displayed higher SPID (the sum of the pain intensity differences), TOTPAR (the sum of the hourly pain relief values), Peak PID (the pain intensity difference) percentage than group placebo and group acetaminophen.

Zhong et al compared the epidural analgesia and oral oxycodone for pain control.^[26]^ Patients in epidural analgesia group received 0.1% ropivacaine + 0.1 μg/mL sufentanil using a PCA machine managed with loading dose of 5 mL, continuous infusion of 8 mL/h, patient-controlled bolus dose of 4 mL, lockout interval of 15 minutes, and maximum dose of 16 mL/h immediately after the peritoneum closed. Patients in oral oxycodone group took 15 mg CR oxycodone in the recovery room every 12 hours for the first postoperative 24 hours, thereafter 10 mg every 12 hours for the next 24 hours. One tablet of paracetamol and tramadol (containing 37.5 mg tramadol and 325 mg paracetamol) was also administrated orally every 6 hours for the postoperative 48 hours. For both groups, breakthrough pain was managed with intramuscular pethidine (50 mg), if analgesia was inadequate, second dose of pethidine was provided. In oral oxycodone group, patients experienced less evoked pain and uterine cramping pain at 6, 12, 24, 36, and 48 hours, and less resting pain at 6, 12, 24, and 36 hours postoperatively. Only 2 patients requested pethidine in epidural analgesia group.

In Dieterich et al study,^[24]^ patients were randomly assigned to received either 20 mg oxycodone at fixed intervals at 2 and 12 hours after CS, or IV PCA piritramide (patient was administered intravenously 1 mg piritramide with a lock out time of 5 minutes, discontinued at 24 hours. The maximum dose was 30 mg. For baseline analgesia, ibuprofen 500 mg Q8h was given for the first day after cesarean. 500 mg ibuprofen and 1 g acetaminophen were offered for rescue medication as needed. Mean VAS scores s of the oxycodone and IV PCA piritramide group were 5.04 ± 2.15/4.35 ± 2.70 (12 hours) and 5.88 ± 2.01/4.85 ± 2.23 (24 hours) (*P* > .05), and the consumption of rescue medication after 48 hours was not different (*P* = .057).

Courtney and colleagues investigated the combination oxycodone–acetaminophen and separately administered oxycodone/acetaminophen for pain control after CS.^[21]^ The drug for the “Combined Medication” group was oxycodone 5 mg-acetaminophen 325 mg 1 or 2 tabs Q4h prn. For the “Separate Medication” group, delivery women were given acetaminophen 650 mg Q4h prn and oxycodone 5 mg or 10 mg Q4h prn. All parturient were prescribed ketorolac Q6h scheduled for 24 hours, and then ibuprofen 600 mg Q6h prn was followed. Inpatient opioid medication use per 12-hour period was calculated to IV morphine milligram equivalents per 12-hour period. Compared with Combined Medication group, Separate Medication group showed fewer morphine milligram equivalents IV morphine per 12 hours (*P* < .05) while using a multiple regression model controlling for covariates. The acetaminophen use per 12-hour period was similar between groups.

Schoenwald et al compared the IR oxycodone and CR oxycodone for post cesarean pain management.^[20]^ The intervention group received IR oxycodone at 8:00 hours (10 mg), 14:00 hours (5 mg), and 20:00 hours (5 mg) on the day after surgery with nurse practitioner intervention. The control group was ordered 10 mg of CR oxycodone commencing at 08:00 hours and continuing per 12 hours for 48 hours. If the pain was uncontrolled, oxycodone or tramadol could be given as needed. For both groups, oral paracetamol (1 g) Q6h was prescribed at 18:00 hours on the day of surgery, and oral ibuprofen (400 mg) QD for 72 hours was also administrated at 08:00 hours on the first postoperative day. Using linear mixed regression models, the rest or on sitting or moving pain scores were similar between groups. The intervention group under nurse practitioner intervention showed less pain interference with less oxycodone consumption (*P* < .05). While considering the perceived control over pain management, no difference between groups was found.

Katja et al compared the oral and intravenous oxycodone for pain relief after CS.^[14]^ Parturient in the IV PCA oxycodone group received an electronic analgesia pump occupied with oxycodone 1 mg/mL, self-controlled bolus dose 2 mg and a lockout interval of 10 minutes. The oral oxycodone group took an oxycodone 5 mg capsule on demand, the maximum dose was 60 mg in 24 hours. As the background medication, patients in both groups were administrated oral oxycodone 10 mg + naloxone 5 mg, ibuprofen 600 mg, and paracetamol 1 g at 1 hour after surgery. Then the oxycodone/naloxone dose was given Q12h, and ibuprofen and paracetamol were given Q8h. There were no statistically differences in numerical rating scale (NRS) pain scores at rest or satisfaction between the groups at 2, 4, 8, and 24 hours after surgery. There were 5 patients in IV PCA oxycodone group and 0 patient in oral oxycodone group suffered severe pain at rest at 24 hours, NRS pain scores at coughing was higher in the oral oxycodone group at 24 hours (*P* < .05). The mean consumption of intravenous oxycodone was 58.2 mg in IV PCA oxycodone group, 48.3 mg (counted equianalgesic dose of oral oxycodone) in oral oxycodone group (*P* < .05).

In other studies, oxycodone was administered intravenously or epidurally. In wang et al investigation,^[18]^ puerperal were randomly divided into 2 groups to received 0.8 mg/kg oxycodone or 0.8 mg/kg morphine using IV PCA device according to the following protocol: diluted in 100 mL, 2 mL/h continuous dose, and a booster dose of 2 mL/15 minutes. The NRS pain scores were similar between the 2 groups at 1, 2, 6, 12, 24, and 48 hours (*P* > .05).

Leong et al compared epidural preservative-free morphine 3 mg or epidural preservative-free oxycodone 3 mg for analgesia after cesarean delivery.^[17]^ Regular paracetamol 1 mg time-domain spectroscopy (TDS) and mefenamic acid 500 mg TDS were administrated. Oral tramadol 50 mg TDS was provided for breakthrough pain upon request. NRS pain score at rest at 2 to 4, 4 to 8, 8 to 12, and 12 to 24 hours, NRS pain score on movement at 4 to 8 and 8 to 12 hours, and NRS pain score for uterus cramping at 24 hours were higher in oxycodone group than in morphine group.

In Jingjing et al research,^[16]^ primiparas were randomly divided into one of the following 4 treatment regimens: group S (sufentanil 100 μg), group OS1 (sufentanil 70 μg, oxycodone 30 mg), group OS2 (sufentanil 50 μg, oxycodone 50 mg), and group O (oxycodone 100 mg) using an intravenous PCA device. The initial button push dose in each group were sufentanil 2 μg, sufentanil 1.4 μg and oxycodone 0.6 mg, sufentanil 1 μg and oxycodone 1 mg, and oxycodone 2 mg, correspondently. The following protocol were: diluted to 100 mL, 1 mL/h continuous infusion, a self-controlled bolus dose of 2 mL, and a lockout time of 15 minutes, maximum dose of 10 mL/h. Flurbiprofen axetil 50 mg was injected intravenously at 0 and 6 hours. NRS of uterine cramping pain and NRS of moving into the sitting position at 6, 12, and 24 hours, and NRS of incision pain at rest at 12 and 24 hours in group O were lower than those in the other treatment groups (*P* < .05). Patients in group O pressed lower number of patient-controlled intravenous analgesia boluses than these in group OS1 and group S at 6, 12, and 24 hours. Patients in group O consumed lower opioid dosage (sufentanil equivalents) than that in group OS1 and group S.

### 3.3. Postoperative side effects

Several included studies reported lower or similar incidences of side effects such as nausea, vomiting, pruritus, and sedation with oxycodone when compared with other medication. In Kathryn et al study,^[22]^ promethazine 25 mg intramuscularly was given Q4h as needed for nausea. Patients developed less nausea at 6 and 24 hours in oral oxycodone group than IV PCA morphine group (*P* < .05), but incidence of emesis was similar between groups at 6 and 24 hours. There was less sedation in the oral oxycodone group at 6 hours (*P* < .05), but no difference at 24 hours (*P* > .05). The drowsiness and pruritus were comparable between groups. In another study by Niklasson et al,^[23]^ there are 3 women in the oral oxycodone group reporting adverse effects (0–24 hours, 3 women felt dizziness) vs 15 women reporting adverse effects (0–24 hours, 9 women felt dizziness, 4 women processed nausea, 1 women felt being tired, 4 women developed itching) in intravenous morphine group (*P* < .05). In study by McDonnell et al,^[25]^ 87% women reporting pruritus in intrathecal morphine group versus 56% women in oral oxycodone group at 24 hours (*P* < .05). In Katja et al study,^[14]^ 16% parturient reporting nausea in IV PCA oxycodone group versus 3% parturient in oral oxycodone group at 4 hours, 10% parturient reporting vomiting in IV PCA oxycodone group versus 2% in parturient in oral oxycodone group at 8 hours (*P* < .05). In Leong et al research,^[17]^ pruritus severity scores were worse for epidural morphine group at 4 to 8, 8 to 12, and 12 to 24 hours postoperatively, but treatment for pruritus was similar between the 2 groups. The need for treatment of PONV, the incidence of flatulence, open bowels and presence of a urinary catheter were similar between groups. No patients suffered from respiratory depression in either group. In Jingjing et al research,^[16]^ nausea was observed in 5 patients in group S, 0 patients in other groups during the first 6 hours after the operation. No significant differences were observed in the incidences of hypotension, hypoxemia, respiratory depression, vomiting, pruritus, Ramsay Sedation Score or dizziness among the 4 groups. Zhong et al found higher incidence of pruritus in the epidural analgesia group than that in oral oxycodone group (*P* < .05).^[26]^ There were no significant differences between groups for the incidence of dizziness, nausea, or vomiting, sedation scores. R. Ffrench et al showed that side effects were common with 71% experiencing at least one in the oxycodone group and 70% in the tapentadol group.^[15]^ There were no serious adverse events reported.

### 3.4. Postoperative recovery

In Niklasson et al study,^[23]^ the time of first bowel movement was faster in oral oxycodone group than that in intravenous morphine group (2.9 ± 1.4 vs 3.6 ± 1.2 day postoperatively), time of stand next to the bed, walking around with help, fully mobilized, discharge from hospital were similar between groups. In Kathryn et al study,^[22]^ ambulation, oral intake were comparable between groups. In study by McDonnell et al,^[25]^ urinary retention was comparable between groups. In Katja et al study,^[14]^ first meal, mobilization, defecation, abdominal distension were comparable between groups. In Zhong et al study,^[26]^ no significance was found in anus exhaustion time, free to mobilization time, or hospital duration between groups.

### 3.5. Patient satisfaction/hospital costs

In study by McDonnell et al,^[25]^ the intrathecal morphine group had higher satisfaction scores than these in oral oxycodone group at 24 hours, but this advantage was no longer present by 48 hours. In Jingjing et al research,^[16]^ number of patients with very satisfactory was higher in group O than in group S, number of patients with neutral was lower in group O than in group S. In Zhong et al study,^[26]^ patients in oral oxycodone group had higher satisfaction scores than in the epidural analgesia group (90.0 ± 9.8 vs 82.0 ± 10.0; *P* < .05). In Dieterich et al study,^[24]^ Leong et al^[17]^ and Katja et al^[14]^ research, there was no difference in general satisfaction with the respective pain management between groups.

In Dieterich et al study,^[24]^ the costs for oxycodone group was 2.98€, expenses in IV PCA piritramide group amounted to 36.73€.

## 4. Discussion

This narrative review revealed that the efficacy of oxycodone in different formulation compared with other interventions of analgesia in CS. Oxycodone showed superior or similar postoperative analgesic efficacy compared with other opioids in various administration, and reduced the need for rescue medication and side effects.

Oxycodone is one of the common opioid analgesics used after CS. Oxycodone has a neutral taste, which make it easily accepted by patients. The oral administration is preferred due to its high maternal acceptability, cheapness and convenience, while the breast milk transfer of oxycodone is low and it is associated with minimal risk to the neonate because of low volumes of breast milk intake.^[27–29]^ In our study, 2 included studies indicate oral oxycodone provide comparable or better pain management than other oral analgesic and reduced the requirement of rescue medication.^[15,19]^ Oxycodone is available as IR and CR tablets or capsules. Previous research showed that CR oxycodone provided similar fast onset of analgesia to IR oxycodone.^[30]^ One included study reported that the intervention group (oral IR oxycodone) under nurse practitioner intervention showed less pain interference with less oxycodone consumption (*P* < .05). But considering the perceived control over pain management, no difference between groups was found.^[20]^

Oral oxycodone has several advantages over the intravenous analgesia postoperatively. Firstly, the bioavailability of oral oxycodone was between 45 and 87%, it is higher than the relative morphine (15–30%).^[30,31]^ Secondly, its fast transportation of blood–brain barrier and ease of administration established the safety and effectiveness of pain control.^[32]^ Previously studies had showed that 30 minutes after oral oxycodone, effective pain treatment can be gained.^[33,34]^ Thirdly, oxycodone, a semisynthetic thebaine derivative µ-opioid receptor agonist, is proved to be more effective than morphine in controlling the visceral pain.^[35]^ Despite gastrointestinal function decrease after CS, early oral intake is proved to be acceptable and is associated with earlier recovery of bowel function.^[36–38]^ Seaton et al reported that oral administration of oxycodone (≤90 mg in 24 hours for up to 3 days) for pain control after CS posed a minimal risk to neonates. Adequate analgesia allows the mother comfortably to feed the newborn.^[18,28]^ In our research, 4 included research found oral oxycodone equipotent or better analgesic effect than intravenous opioids.^[14,22–24]^

Neuraxial analgesic techniques are commonly recommended for post-cesarean analgesia. Two included studies revealed that oral oxycodone produced comparable or better postoperative pain relief after CS to neuraxial analgesia.^[25,26]^ Fewer data are available on neuraxial use of oxycodone. One included research showed NRS pain scores on movement and cramps were higher in epidural oxycodone group than in epidural morphine group, although maternal satisfaction of analgesia was similar between groups.^[17]^ Intravenous oxycodone is not yet frequently utilized for postoperative analgesia, but recent clinical studies suggest that intravenous oxycodone produced better pain relief than morphine or fentanyl.^[39,40]^ In our review, intravenous oxycodone showed superior analgesic efficacy than intravenous sufentanil in terms of uterine contraction pain, possibly due to oxycodone’s к-opioid receptor agonist properties.^[16]^ Based on the time to postoperative lactation initiation (55–56 hours) and the elimination half-life of oxycodone (2–3 hours), there would be extremely low concentration of oxycodone in breast milk.^[16]^

Multimodal analgesic approach, which involve the combination of opioid and nonopioid therapies, are commonly recommended for post-cesarean analgesia.^[13]^ The combination of opioid and nonopioid drugs with different mechanisms of action can assist in pain relief postoperatively with reduced opioid consumption and side effects. In this review, several studies used the opioids and NSAIDs for the pain management.^[14,22,26]^

Oxycodone exerts side effects similarly to opioid agonists. Opioid-related adverse effects including CNS depression and gastrointestinal dysfunction are partly determined by the dose and route of administration.^[11,41]^ This narrative review that there is still a certain prevalence of adverse effects associated with oxycodone such as nausea, vomiting, dizziness and pruritus. But incidence of side effects in oxycodone group was lower or similar to the other analgesic group.^[14,15,17,22,23,25,26]^ The Enhanced Recovery After Surgery for postoperative care in cesarean is aimed to enhance recovery, reduce length of stay in hospital, reduce the cost and reduce complications.^[42]^ One study in this review reported first bowel movement was earlier in oxycodone group than another opioid group.^[23]^ First meal, mobilization, defecation, abdominal distension and discharge from hospital were similar between oxycodone and other opioid group.^[14,22,23,25,26]^ One research showed lower cost in oxycodone group than other opioid group.^[24]^

There are several limitations in this narrative review. Firstly, we do not register the review in Chinese Clinical Trial Registry. Secondly, the pain management plans are different in each included studies, it’s hard to calculate the exact analgesic doses in terms of a lack of consistent equianalgesic dose ratio between oxycodone and other opioids. Furthermore, this narrative review can have considerable bias due to the heterogeneity of pain management techniques and pain assessment ways. Finally, we didn’t describe evidence included in the review (e.g., study risk of bias, inconsistency, and imprecision) in detail. Nevertheless, a broad overview of the clinical utility of oxycodone for pain management is provided for clinicians.

## 5. Conclusion

Oxycodone can be successfully used also for postoperative analgesia after CS and was associated with comparable levels of side effects. Compared other opioids in various administration, oxycodone may provide superior or similar analgesia, and reduced the need for rescue medication; in many studies, oxycodone was associated with fewer adverse effects, such as nausea and vomit. Further randomized, controlled trials in this area are needed to provide the best evidence base for practice.

## Author contributions

**Data curation:** Qingqing Pei, Zhiyou Peng.

**Formal analysis:** Hongmei Xuan.

**Methodology:** Hongmei Xuan.

**Writing – original draft:** Qingqing Pei.

**Writing – review & editing:** Zhiyou Peng.
